# Estimation of the number of motor units in the human extensor digitorum brevis using MScanFit

**DOI:** 10.1371/journal.pone.0302214

**Published:** 2024-04-26

**Authors:** Cliff S. Klein, Hui Liu, Yuan Xiong

**Affiliations:** Guangdong Work Injury Rehabilitation Center, Guangzhou, Guangdong, China; Sultan Qaboos University College of Medicine and Health Science, OMAN

## Abstract

**Objective:**

Our aim was to determine the number and size parameters of EDB motor units in healthy young adults using MScanFit, a novel approach to motor unit number estimation (MUNE). Since variability in MUNE is related to compound muscle action potential (CMAP) size, we employed a procedure to document the optimal EDB electromyographic (EMG) electrode position prior to recording MUNE, a neglected practice in MUNE.

**Methods:**

Subjects were 21 adults 21–44 y. Maximum CMAPs were recorded from 9 sites in a 4 cm^2^ region centered over the EDB and the site with the largest amplitude was used in the MUNE experiment. For MUNE, the peroneal nerve was stimulated at the fibular head to produce a detailed EDB stimulus-response curve or “MScan”. Motor unit number and size parameters underlying the MScan were simulated using the MScanFit mathematical model.

**Results:**

In 19 persons, the optimal recording site was superior, superior and proximal, or superior and distal to the EDB mid-belly, whereas in 3 persons it was proximal to the mid-belly. Ranges of key MScanFit parameters were as follows: maximum CMAP amplitude (3.1–8.5 mV), mean SMUP amplitude (34.4–106.7 μV), mean normalized SMUP amplitude (%CMAP max, 0.95–2.3%), largest SMUP amplitude (82.7–348 μV), and MUNE (43–103). MUNE was not related to maximum CMAP amplitude (R^2^ = 0.09), but was related to mean SMUP amplitude (R^2^ = -0.19, P = 0.05).

**Conclusion:**

The EDB CMAP was highly sensitive to electrode position, and the optimal position differed between subjects. Individual differences in EDB MUNE were not related to CMAP amplitude. Inter-subject variability of EDB MUNE (coefficient of variation) was much less than previously reported, possibly explained by better optimization of the EMG electrode and the unique approach of MScanFit MUNE.

## Introduction

The motor unit, comprising the motoneuron and all muscle fibers it innervates, is the final common pathway through which the nervous system controls force. Denervation-induced loss of motor units in aging, disease, or injury may significantly impact force control and force generating capacity [1], depending on the extent of compensatory reinnervation. It would be clinically useful to have a tool that quantifies the exact number of motor units in a muscle, but no such tool exists. However, the number of functioning motor units can be estimated using electrophysiological techniques, referred to as motor unit number estimation (MUNE) [2, 3].

McComas first introduced MUNE by recording evoked extensor digitorum brevis (EDB) electromyographic (EMG) responses in response to manual incremental stimulation of the peroneal nerve [4]. MUNE is based on a simple premise; the number of motor units can be estimated by dividing maximal compound muscle action potential (CMAP) size by the mean surface-recorded motor unit potential (SMUP) size. Investigators have since introduced refinements including computer-aided stimulation or multiple point stimulation MUNE and alternative approaches such as spike-triggered averaging MUNE, statistical MUNE, motor unit number index (MUNIX) and CMAP scan recordings (see Table 2).

MScanFit is a newer approach to MUNE [5]. In this, supramaximal to subthreshold stimulation is applied to the motor nerve to produce a detailed stimulus-response curve or CMAP scan [6]. A mathematical model is applied to characterize numbers, thresholds, and SMUP amplitudes of all units underlying the CMAP scan or “MScan”. MScanFit MUNE has been found to be reproducible and sensitive at detecting motor unit loss in different muscles [7–13]. However, no one has used it to examine motor unit number in the EDB, although other methods have been employed.

McComas chose the EDB for MUNE because it offers certain advantages over other muscles [4]. It is relatively isolated on the dorsum and is the only muscle in the region innervated by the deep peroneal nerve. Thus, contamination of the EDB CMAP from other signals is less compared to other muscles [14]. The EDB is also flat at less than 10 mm thick [15] with a skinfold less than 3.3 mm [4]. Given the thinness and close proximity of the EDB to the recording surface, most of it’s SMUPs likely contribute to the CMAP maximum.

EMG electrode position influences the maximum CMAP amplitude (or CMAP maximum). This is clearly demonstrated by documenting CMAP maximums at different active (E1) electrode positions (i.e., CMAP mapping) [16, 17]. The EDB CMAP maximum has been mapped by only a few investigators [4, 17, 18], all of whom used unconventional recording electrodes, but MUNE was assessed in only one of these studies [4]. McComas and colleagues used silver foil strip electrodes [4]. The end-plate zone was found to lay across the muscle perpendicular to the extensor hallucis longus tendon (S1 Fig in S1 Appendix). Currier examined 12 females with a custom block electrode aligned parallel with the EDB long axis, but the exact positioning was not disclosed [18]. Finally, the EDB, together with the extensor hallucis brevis, CMAP maximum was mapped in 7 subjects using a multielectrode array [17].

Suboptimal placement of the active electrode away from the end-plate zone may impact MUNE [19, 20]; variability in MUNE and CMAP maximum are often positively correlated in groups of individuals [10, 21, 22]. In theory, suboptimal placement may reduce mean SMUP size relatively more than CMAP maximum resulting in a higher MUNE, or the opposite pattern may occur (S1 Fig in S1 Appendix). The EDB CMAP is particularly sensitive to electrode position based on CMAP mapping [17]. Thus, the average area where CMAP amplitudes were greater than 80% of the maximum amplitude was relatively small in the EDB (1.7 cm^2^) compared to foot sole muscles (18.4 cm^2^) during stimulation of the peroneal and tibial nerves, respectively.

Documenting the optimum EMG electrode position and detailed procedures for optimization are rarely provided in MUNE studies [4]. The recommended practice is to record CMAP maximums at several different positions. The optimum site is deemed to be the position with the largest CMAP and shortest rise time. Often, it is unclear in published works whether this procedure was followed. Even when followed in studies of EDB MUNE, electrode positions relative to anatomical landmarks and the regions examined were not disclosed [21, 23]. A procedure to locate and document the optimal EMG recording position would seem to be a prerequisite in MUNE investigations as it would be expected to increase precision and decrease variability of MUNE values.

Here we have conducted a physiological study, combining CMAP mapping and MUNE measurements. The purpose was to estimate the number and size parameters of EDB motor units in healthy young adults using MScanFit MUNE after first documenting the optimal EMG electrode position in each subject. Our results indicate that inter-subject variability of EDB MUNE values was less than previously reported, possibly explained by better electrode optimization and the unique approach of MScanFit MUNE.

## 2. Materials and methods

### 2.1 Subjects

Participants were a convenience sample of 21 adults (6 females and 15 males), recruited from hospital staff or students between April 1 2019 and July 31, 2022. Their age ranged from 21–44 y (mean, 32.2 ± 5.9 y). Height and weight were 168.2 ± 7.1 cm and 63.9 ± 10.9 kg. Exclusion criteria were a history of systemic disease or neuromuscular disorders, symptoms of peroneal nerve neuropathy, or abnormal peroneal nerve conduction study results. The study was approved by the hospital’s Medical Ethics committee (no. AF/SC-07/2019.06). All participants completed and signed a written informed consent form approved by hospital’s Medical Ethics committee in accordance with the Declaration of Helsinki prior to testing.

### 2.2 EDB CMAP mapping and MScan recordings

#### 2.2.1 Experimental set-up

Participants were seated with the foot stabilized on a custom platform with a velcro strap across the toes and the knee flexed between 90 and 110° (180° equals full extension) and the ankle plantarflexed 120°. A seated rather than supine position was chosen to be consistent with our EDB studies in patients who were examined with the same platform while confined to their wheelchair. Skin over the stimulation and recording sites were well prepared using a scrub gel and alcohol. EDB activity during nerve conduction studies and MUNE experiments was recorded by silver-silver chloride disposable electrodes (1 cm diameter circular snap button in a 2.2 cm x 2.2 cm pre-gelled/adhesive cloth backing, Kendall H69P, Natus Neurology, WI, USA). The reference (E2) electrode was positioned at the base of the 5th toe and the active (E1) electrode at the established optimal site over the EDB (see section 2.2.3). A ground electrode (1 cm^2^ metal plate) was placed on the dorsum. Skin temperature was recorded by a thermistor on the dorsum (Omega Engineering Inc., Stamford, CT, USA), but was not controlled. During the experiments, subjects were asked to remain relaxed and refrain from moving or talking. This set-up was used for mapping EDB CMAPs and the subsequent MScan recording.

#### 2.2.2 Peroneal nerve stimulation and EDB EMG recording

The peroneal nerve was stimulated by a 2 disc bar electrode (3 cm spacing between discs) filled with conductive gel. It was positioned inferior to the fibular head with the cathode distal, and secured in place with an elastic strap [24]. The pulse duration was set to 1 ms to be consistent with our patient studies. In some patients, the maximal output of the stimulator (50 mA) was insufficient to evoke a supramaximal CMAP when pulse duration was less than 1 ms. Preliminary experiments revealed that MScan responses evoked by peroneal nerve stimulation at the ankle were uncomfortable; thus, stimulation at the fibular head was used for definitive MScan recordings.

Stimulation and recording were controlled by QTracS software (© Professor H. Bostock, Institute of Neurology, London). Pulses generated by computer were converted to current via a constant current stimulator (DS5, Digitimer Ltd., Welwyn Garden City, Hertfordshire, UK). EMG activity was amplified (x500) and bandpass filtered (10 Hz to 3 kHz) (Astro-Medical, model P511, West Warwick, Rhode Island). A noise eliminator (Hum Bug 50/60 Hz, Digitimer Ltd) removed line frequency noise. EMG was digitized at a sampling rate of 10 kHz with a 16-bit converter (NI-USB6221; National Instruments; Austin, Texas).

#### 2.2.3 CMAP mapping

**Fig 1:**
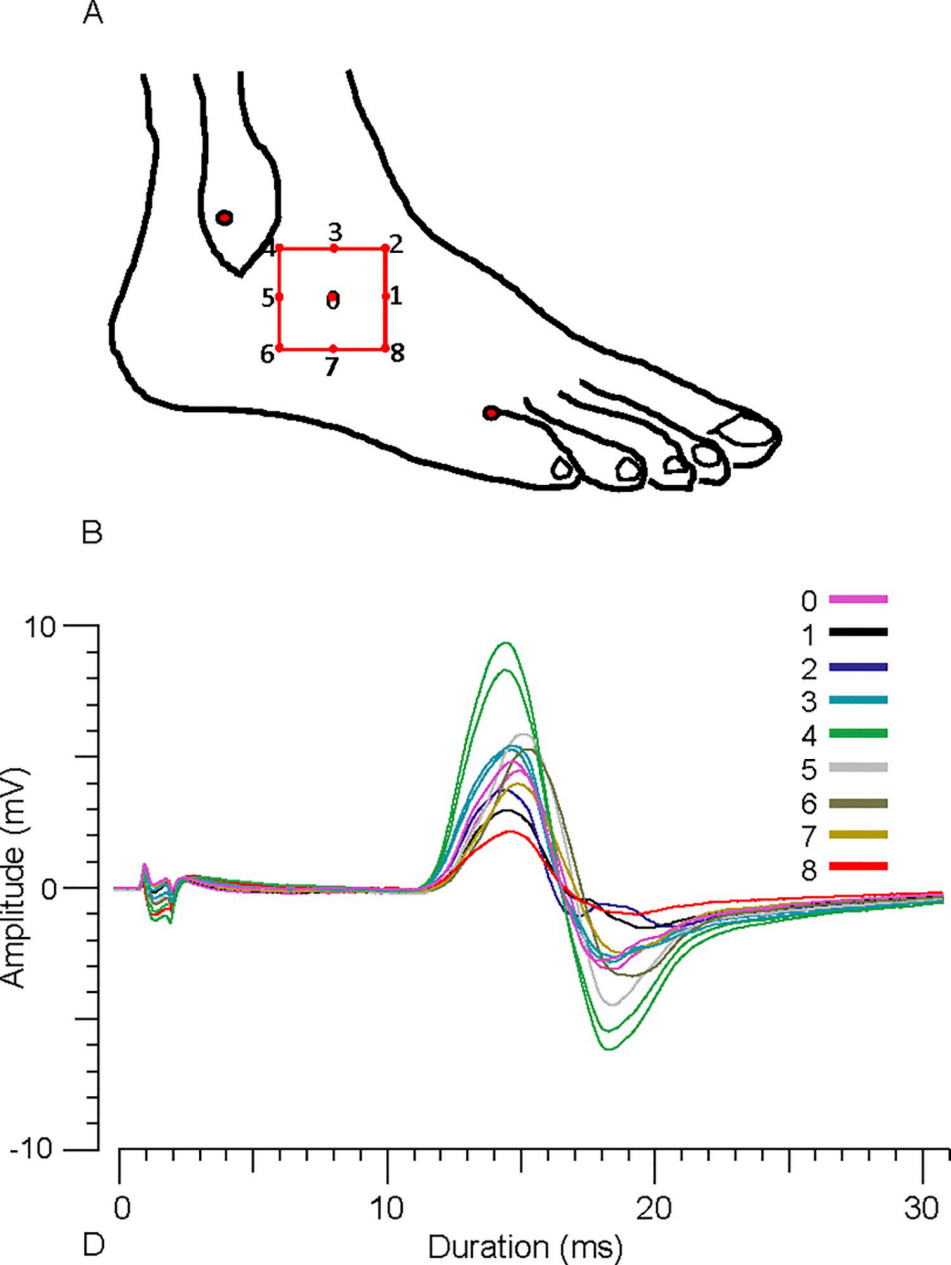
CMAP mapping. **(A)** Positions of the active EMG electrode used for mapping maximal CMAPs from 9 sites (site 0 to site 8). Site 0 is located at the approximate EDB mid-belly, and sites 1 to 8 are located at the designated target positions about the perimeter of a 2 cm x 2 cm square drawn on the skin. Also shown are 2 ink marks (one over the lateral malleolus and the other at the 5^th^ toe cleft) for determining the one-third distance to the EDB mid-belly **(B)** Overlay of CMAP waveforms in one participant. Single maximal CMAPs from sites 0 to 8, and repeat recordings from sites 0, 3 and 4, are displayed. In this case, site 4 was the optimal recording site.

To minimize MUNE variability that may arise from suboptimal electrode placements, we employed a relatively simple standardized procedure to position the active electrode at a site that resulted in the largest CMAP negative peak amplitude. Specifically, maximal CMAPs were recorded from 9 pre-determined EDB sites. The site that produced the largest amplitude was deemed to be the optimal recording site and was used in the MScan recording. First, with the foot on the plate, an ink mark was made over the EDB, one-third the distance from the lateral malleolus to the dorsal cleft between the 4^th^ and 5^th^ toes (site “0”, Fig 1A). Pilot experiments revealed that the one-third distance corresponded to the approximate EDB mid-belly, at least in those persons with a prominent EDB lump at rest. Next, a 2 cm x 2 cm square was drawn on the skin using a plastic template, with site “0” at it’s center. The maximal CMAP at site 0 was recorded by stimulating the peroneal nerve at the fibular head with maximal and then supramaximal stimuli. Using the same supramaximal intensity, single maximal CMAPs were then recorded at each of the remaining 8 sites about the square, with a 15 s delay between each stimulus. After recording from the 9 sites, repeat recordings were made from a few sites in some persons for examination of repeatability.

CMAPs from the 9 sites were recorded with a hand-held EMG electrode. This was a standard lead wire and snap button portion of the electrode with the cloth backing and gel removed. However, we added a thin film of gel to the button because pilot experiments revealed that the EMG baseline may otherwise shift. The electrode was moved in ~1 cm steps about the square’s perimeter in a counter-clockwise direction; from site 0 to site 1, site 1 to site 2 etc. Preliminary experiments during which recordings were made with the electrode moved in a clockwise direction did not change the results. Throughout recordings, CMAP waveforms and amplitude values were monitored on-line. For subsequent MScan recordings, a disposable electrode was positioned at the established optimal recording site.

#### 2.2.4 MScan recording

MScans were recorded using the subroutine of the QtracS TRONDNF protocol (Qtrac-S, version 25/01/2019) [5, 7]. First, the maximum CMAP amplitude was recorded; stimuli were applied at the fibular head at a frequency of 2 Hz as the stimulus current was manually increased (2% steps) until the CMAP maximum was reached, and then increased further by ~ 20% (supramaximal stimuli). A pre-scan of 20 supramaximal CMAPs were then recorded. The stimulus intensity was then automatically reduced in 0.2% steps until there was no response, after which 20 additional stimuli were applied to record a clean baseline.

### 2.3 Peroneal nerve conduction study

Maximum conduction velocity was derived from EDB maximum CMAPs evoked by proximal (stimulation at the fibular head, recorded during the MScan) and distal stimulation of the peroneal nerve. For stimulation at the ankle, the cathode was applied 8 cm proximal to the active EMG electrode, with the anode proximal. These responses were recorded after the MUNE recording using the same electrodes. Stimulation was not applied in the popliteal fossa to test for entrapment neuropathy. Stimuli were controlled by the QtracS software and applied as described previously for establishing CMAP maximums during stimulation at the fibular head.

### 2.4 Sural nerve conduction study

A sural nerve conduction study was done according to established methods [24]. The stimulus-response component of the QtracS software for sensory nerve action potentials was used to record sural nerve action potentials. A standard bar electrode (3 cm spacing between discs) was placed behind the lateral malleolus to record antidromic sensory responses. Stimulation (0.2–0.5 ms pulse duration) was applied by another bar electrode; the cathode was placed in the posterior midline of the lower leg 14 cm proximal to the active EMG electrode, with the anode proximal.

### 2.5 Accessory deep peroneal nerve

The accessory deep peroneal nerve is a relatively common variant innervation of the EDB, occurring in ~ 20% of tested legs [25]. In the present study, we only tested for its presence when the maximum EDB CMAP amplitude was found to be larger when evoked by stimulation at the fibular head compared to stimulation at the ankle (the opposite of the usual pattern). We found this opposite pattern in only 1 of the 21 subjects, and presence of an accessory nerve was confirmed when stimulation was applied posterior to the lateral malleolus (S2 Fig in S1 Appendix).

### 2.6 Data analysis

#### 2.6.1 MScanFit MUNE

The same investigator (CSK) performed all analysis. A mathematical model of the QtracP program (MScanFit-2, version 24/01/2023) was used to simulate recorded MScans and derive MScanFit MUNE and size parameters. The size parameters were (1) maximum CMAP negative peak amplitude (mV); (2) smallest SMUP amplitude (μV); (3) largest SMUP amplitude (μV and %CMAPmax); (4) mean SMUP amplitude (μV and %CMAPmax); (5) A50 (μV), motor unit size at the 50% mark of the cumulative amplitude, and (6) N50, number of motor units that make up the top 50% of the cumulative amplitude. The model consists of *N* units, each described by 3 parameters; threshold, amplitude, and relative spread of threshold. Two additional parameters account for variability in baseline noise (i.e., the post scan period free of evoked responses) and peak CMAP (i.e., the pre-scan period of 20 CMAPs).

The MScanFit-2 settings were used for modeling the MScan. The minimum unit size was set to the greater of the CMAP amplitude/300, and 1/5th of the mean unit size. MUNE values have been found to be less dependent on CMAP maximum using MScanFit-2 compared to the original MScanFit-1 options [11, 22]. First, a flat section of the pre and post-scan periods, where signal fluctuation was minimal, was set manually. Then, a preliminary model of the MScan was produced based on the recorded variance and slope of stimulus-response points along sections of the scan. The program then applied a number of refinements, altering the number and properties of the modeled units, to minimize the discrepancy (or error score) between the recorded and modeled responses. The equations underlying the model are available in the original report’s supplement [5].

#### 2.6.2 Nerve conduction studies

Peroneal nerve conduction velocity was equal to the distance between the cathode at the knee and ankle divided by the difference between CMAP onset latency evoked by knee and ankle stimulation. For the sural nerve recordings, the maximum peak-to-peak-amplitude of a single response was determined.

#### 2.6.3 Statistical analysis

Statistical analysis of the MScanFit data was done using the QtracP program. Data for all presented MScanFit parameters were found to be normally distributed based on the Lilliefors test of normality, thus permitting parametric analysis. Pearson correlation coefficients were applied to examine relationships between measures. Values are provided as means ± SD, and differences were considered significant when P < 0.05.

## 3. Results

### 3.1 CMAP mapping

Maximum CMAPs recorded at sites 0 to 8 are presented for one representative participant (Fig 1B, also S3 and S4 Figs in S1 Appendix). Waveforms were biphasic (negative-positive) over the 9 sites. In this subject, the largest amplitude occurred at site 4 (8.4 mV). Amplitudes at sites 0 to 8, expressed as a percentage of site 4 amplitude, were 55.3%, 37.1%, 46.1%, 64.2%, 100%, 72%, 63.8%, 48.5%, and 25.6%, respectively. Repeat recordings from sites 0, 3, and 4 (after first recording from all 9 sites) are also displayed for this subject. The amplitude of the first recording was within 12% of the repeat recording at each of the 3 sites; site 0, 4.6 mV vs. 4.8 mV; site 3, 5.4 mV vs. 5.5 mV; site 4, 8.4 mV vs. 9.4 mV, respectively.

The optimal recording site was located mostly superior to the mid-belly at site numbers 2 (N = 1), 3 (N = 4), or 4 (N = 8) along the square’s upper perimeter, or proximal to the mid-belly at site 5 (N = 3). In the remaining 5 subjects, 2 adjacent sites were deemed to be optimal because CMAP amplitudes at these sites were within 5% of each other; at sites 2 and 3 (N = 1), 3 and 4 (N = 3, S4 Fig in S1 Appendix), or 4 and 5 (N = 1). The CMAP amplitudes of the 2 most adjacent sites to the optimal site ranged from the 45% to 93.1% of optimal site amplitude (mean of these 2 sites; 76.4 ± 7.4% of optimal site amplitude), excluding sites ≥ 95% of the optimal site amplitude. The mean maximum CMAP amplitude of the first recording was within 1.6 ± 4.1% (range, -14.9% to 13.3%, N = 8) of the repeat recording at the same optimal site.

### 3.2 Nerve conduction study and MScanFit

Laboratory temperature ranged from 22° to 24° (mean, 23.1 ± 0.6°) and skin temperature ranged from 27.4° to 32.3° (mean 29.6 ± 1.3°). All subjects tolerated the nerve conduction study and MScan recording without incident.

**Fig 2:**
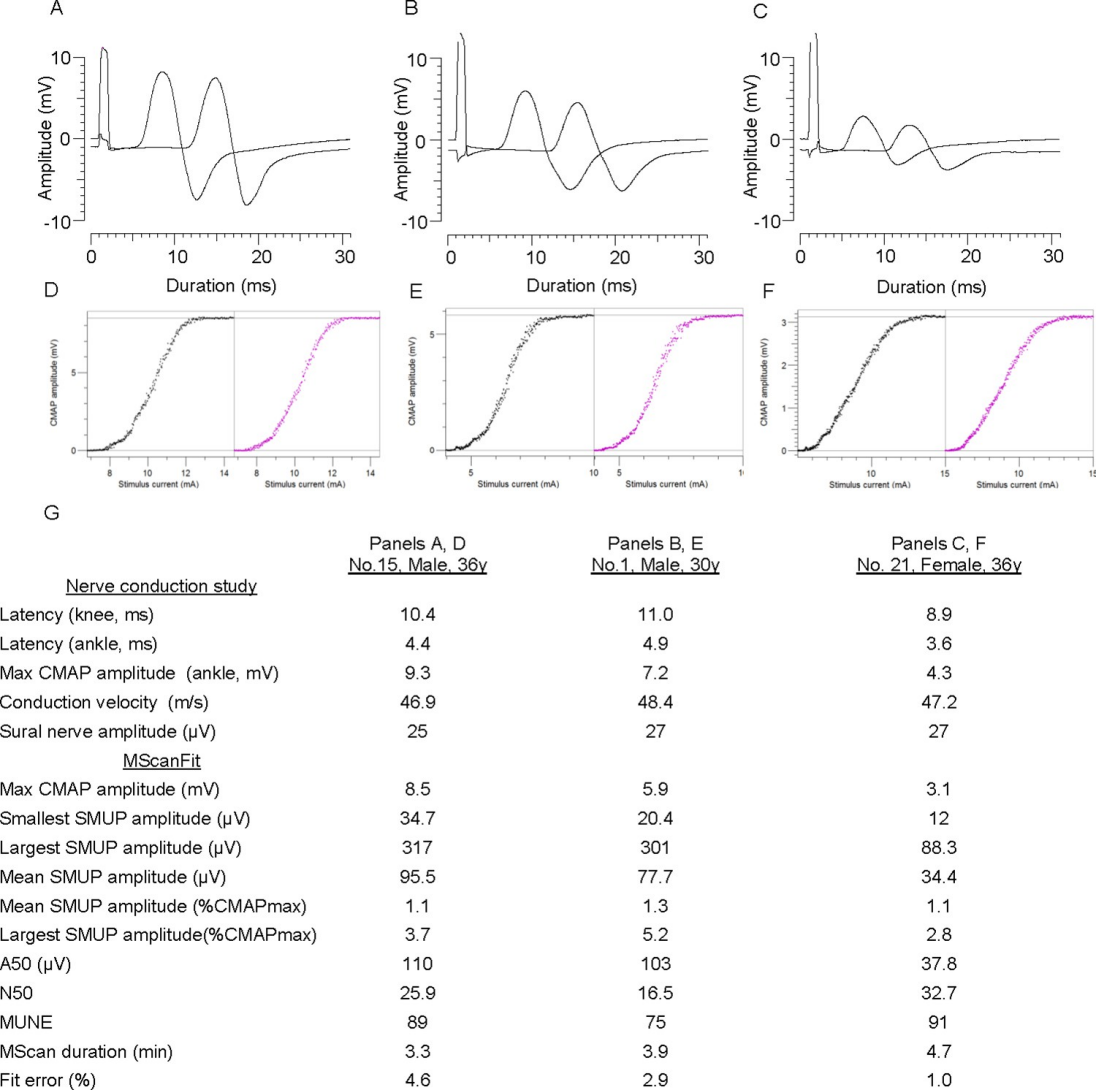
Maximum CMAP waveforms, MScans, nerve conduction and MScanFit results in 3 subjects. **(A, D)** Male 36 y. **(B, E)** Male 30 y. **(C, F)** Female 36 y. Maximum CMAPs evoked by nerve stimulation at the knee and ankle are overlayed in 30 ms windows in A, B, and C. **(D-F)** MScan recording (left) and corresponding modeled MScan (right) for each person. **(G)** Nerve conduction and MScanFit study results in the 3 subjects.

Representative maximum CMAPs evoked by stimulation at the fibular head and ankle are shown for 2 males (one of whom had the largest CMAP of the 21 subjects), and 1 female (who had the smallest CMAP) (Fig 2A–2C). Also shown side-by-side are their recorded and modeled MScans (Fig 2D–2F). All 3 had smooth S-shaped MScans typical of healthy adults less than 60 y of age, indicating little to no large discontinuities or gaps in the stimulus-response curve. Nerve conduction study and MScanFit results for these 3 persons are displayed in Fig 2G. Like all subjects, nerve conduction study results were normal. Group means (ranges) of key parameters are shown in Table 1.

**Table 1:** Nerve conduction study and MScanFit results for all subjects.

Nerve conduction study	
Latency (knee, ms)	9.9 ± 0.9 (8.2–11.8)
Latency (ankle, ms)	4.4 ± 0.6 (3.1–6.1)
Max CMAP amplitude (ankle, mV)	6.1 ± 1.6 (3.0–9.3)
Conduction velocity (m/s)	49.5 ± 3.3 (44.3–56.1)
Sural nerve amplitude (μV)	27.1 ± 9.5 (15–46)
MScanFit	
Maximum CMAP amplitude. (mV)	5.2 ± 1.5 (3.1–8.5)
Smallest SMUP amplitude (μV)	21.9 ± 7.1 (12.0–35.6)
Largest SMUP amplitude (μV)	202.5 ± 78.5 (82.7–348)
Mean SMUP amplitude (μV)	67.6 ± 19.0 (34.4–106.7)
Mean SMUP amplitude (%CMAPmax)	1.32 ± 0.31 (0.95–2.3)
Largest SMUP amplitude. (%CMAPmax)	4.0 ± 1.5 (2.0–9.7)
A50 (μV)	76.6 ± 23.2 (37.8–120)
N50	24.6 ± 6.1 (12.8–34.3)
MUNE	78.5 ± 15.2 (43–103)
MScan duration (min)	4.5 ± 1.0 (3.2–7.6)
Fit error (%)	3.0 ± 1.4 (1.0–5.9)

Values are means (SD) and range; CMAP, compound

muscle action potential; SMUP, surface recorded motor

unit action potential; MUNE, motor unit number estimation.

### 3.3 Correlations between MScanFit parameters

**Fig 3:**
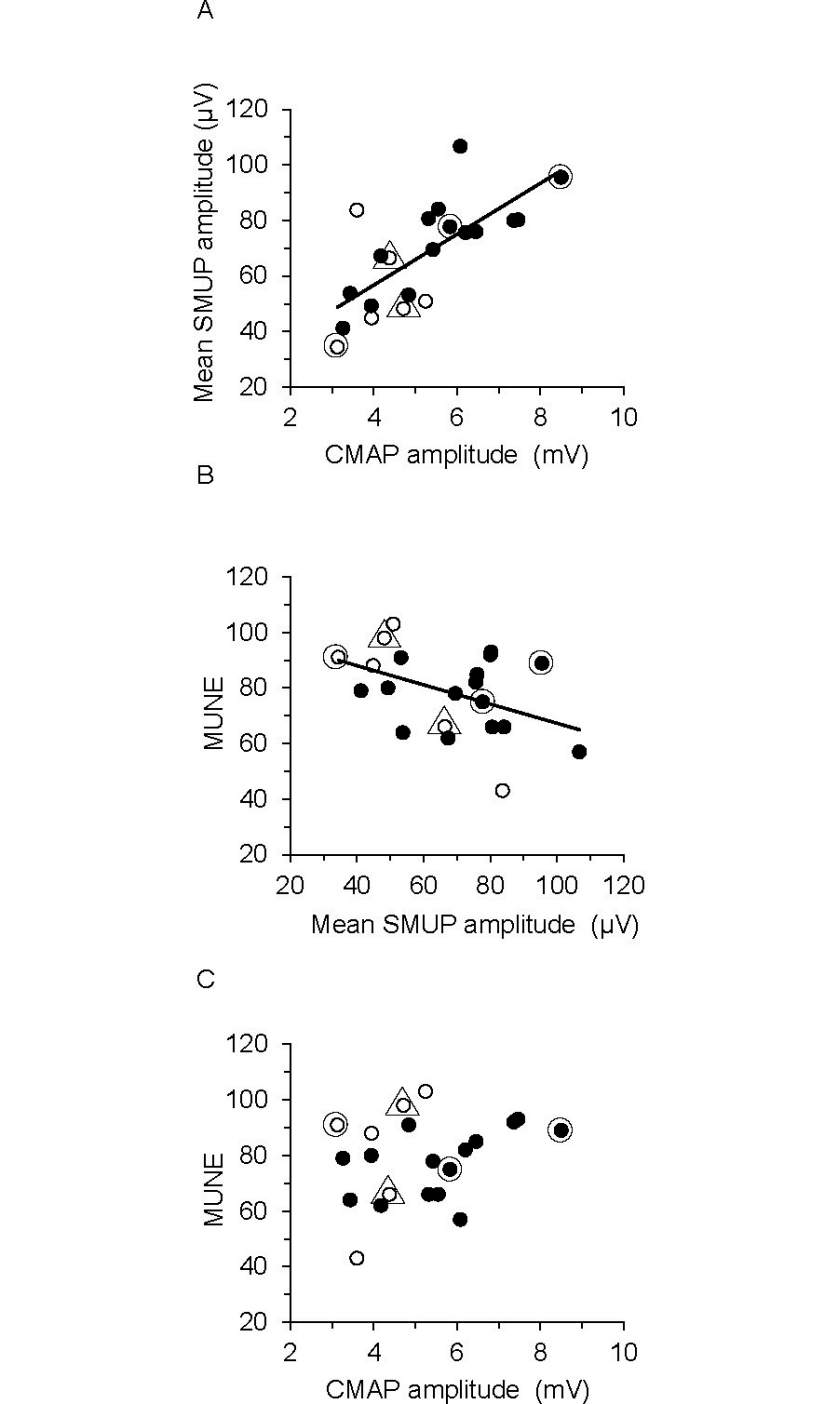
Correlations between MScanFit parameters in all subjects. Data points for males are black symbols (N = 15) and females are white symbols (N = 6). **(A)** SMUP amplitude was positively correlated with maximal CMAP amplitude. **(B)** MUNE was negatively correlated with SMUP amplitude. **(C)** MUNE was not significantly correlated with maximum CMAP amplitude. Lines of best fit are shown only for correlations that were statistically significant. Circled data points correspond to the 3 subjects in Fig 2. Data points surrounded by a triangle are two subjects with similar CMAP maximum but different MUNE.

Correlations between select MScanFit parameters in all subjects were determined. Individual mean SMUP amplitude and maximum CMAP amplitude were correlated (R^2^ = 0.50, P = 0.003, Fig 3A). MUNE was weakly correlated with mean SMUP amplitude (R^2^ = -0.19, P = 0.05, Fig 3B). Also, MUNE was not significantly correlated with maximum CMAP amplitude (R^2^ = 0.09, P = 0.18, Fig 3C).

## 4. Discussion

### 4.1 EDB CMAP mapping

We documented the optimal active EMG electrode position prior to the MScan in each participant using a simple standardized procedure. Our intention was to introduce a quick CMAP mapping method that can be easily adapted by others for research or clinical purposes with conventional electrodes. In previous studies of MUNE or nerve conduction, the active electrode was often placed over the EDB mid-point where most end plates were assumed to be located (i.e., the “motor point”). However, the EDB may not be readily palpable or visible at rest. Furthermore, the optimal site may be off-center and differ between persons [17], and this was confirmed in the present study.

Our findings and others [4, 17] indicate that the optimal EDB recording site varies between individuals. However, we showed that it was commonly located on the square’s upper perimeter, superior to the mid-belly. We also found that moving the electrode 1 cm from the optimal site reduced the CMAP maximum by up to 50%. This degree of EDB CMAP sensitivity to electrode position is greater than in one previous report [18], but is consistent with a more comprehensive mapping study [17]. In the latter, the map origin was one-third the distance from the lateral malleolus to the dorsal cleft between the 3^rd^ and 4^th^ toes, whereas it was the distance to the cleft between the 4th and 5^th^ toes in the present study. Consequently, our origin was probably located about 1 cm inferior to their origin. After accounting for this difference, the optimal sites in the present study are similar to their EDB high amplitude zones [17].

The CMAP maximum of a given muscle may vary widely between healthy young adults, likely reflecting biological variability and methodological factors [17, 22]. There was a 3-fold range in CMAP maximum in the current study. Because we optimized electrode position, electrode proximity to the innervation zone was probably not a primary determinant of large inter-subject differences in CMAP maximum. The more likely primary determinants were differences in muscle anatomy such as muscle size [15] and differences in temporal dispersion of nerve fiber action potentials [17].

### 4.2 Correlations between MScanFit parameters

MScanFit estimates the number of motor units and their size parameters based on modeling unitary axon responses embedded in the MScan [5]. This contrasts with traditional MUNE methods that rely on recording “all-or-nothing” or successively incremental responses of a small sample of low threshold motor units [4, 26]. In the MScanFit MUNE model output, the sum of all SMUP amplitudes is equal to the maximal CMAP amplitude; thus, a correlation between mean SMUP amplitude and CMAP maximum was not surprising (R^2^ = 0.5, Fig 3A). Presumably, the variability in mean SMUP amplitude between persons is partially explained by differences in innervation ratio and mean muscle fiber area; both are primary determinants of maximal tetanic force of healthy and reinnervated motor units [27]. Individuals with relatively smaller number of motor units might be expected to have relatively larger mean SMUP amplitude (R^2^ = 0.19, Fig 3B), analogous to findings in patients with denervation. Although there is dearth of data on this issue in healthy controls, some reported significant negative correlations between EDB MUNE and SMUP amplitude (area) in patients with diabetes or multiple sclerosis (R = -0.38 to -0.56) [28, 29]. The negative correlation in the patients implies that motor units are enlarged via collateral reinnervation in response to motor unit loss.

MUNE and CMAP maximum were not correlated, despite the near 3-fold range of values in both (Fig 3C). Others reported a similar lack of correlation between these measures when data were analyzed using MScanFit-2 [11, 22], although significant correlations were reported based on MScanFit-1 analysis [7, 22, 30]. Our findings are consistent with the notion that CMAP maximum alone is not a reliable indicator of the number of motor units in a muscle [31]. Given the anatomical advantages of the EDB as a model for MUNE, we can speculate that large inter-subject differences in CMAP maximum or MUNE mostly reflect real biological differences rather than experimental error. Thus, some persons may have large differences in CMAP maximum, yet have similar MUNE (Fig 3C, i.e., circled smallest and largest CMAP maximum). In this scenario, small CMAPs could reflect a population of relatively smaller amplitude SMUPs indicative of smaller innervation ratios and muscle fibers, whereas large CMAPs would reflect the opposite (Fig 3A). Other persons may have similar CMAP maximum, yet have large differences in MUNE (Fig 3C, i.e., data points surrounded by a triangle). In this scenario, the smaller MUNE muscle could contain relatively larger amplitude SMUPs, whereas the larger MUNE muscle could contain relatively smaller amplitude SMUPs (Fig 3B).

### 4.3 Comparisons with healthy young adult EDB MUNE literature

All electrophysiological motor unit counting methods provide only estimates of unit number and size parameters. Thus, differences in MUNE values between persons/groups or over time provides the greatest utility, rather than relying on absolute values. Nevertheless, comparisons of published healthy adult (mostly < 60 y) EDB CMAP maximums, SMUP amplitudes, and MUNEs may provide some insight into the possible reasons for differences between studies (Table 2).

**Table 2:** Estimation of numbers and size parameters of extensor digitorum brevis motor units in young adults reported in the literature.

Authors	N, Age (y)	Method	Max CMAP amplitude (area)	Mean SMUP amplitude (area)	Mean SMUP amplitude (% CMAPmax)^6^	Motor unit number
McComas et al. 1971 [4]	N = 41 (10♀) 4–58 y	MIS	5.4 mV^3^ ^.^	30 μV	0.55 ± 41	199 ± 60^7^ (121–583) CoV = 30.1%
Ballantyne et al. 1974 [32]	N = 39 35 ±14 y	MIS	-	-	-	197 ± 49^8^ (125–311) CoV = 24.9%
Ballantyne et al. 1974 [33]	N = 20 38 ± 14 y	MIS	-	56.4 ± 28.5 μV	-	-
Panayiotopoulos et al. 1975 [34]	N = 9	MIS	6.3 ± 1.5 mV	15.9 ± 12 μV	0.25	411 ± 125^8^ CoV = 30.4%
Panayiotopoulos et al. 1976 [35]	N = 39 (13♀) 34 ± 12 y 13–60 y	MIS	-	17.4 ± 16.6 μV	-	366 ± 120^8^ CoV = 32.7%
Weir et al. 1980 [29]	N = 27 46.9 ± 12.4 y	MIS	24.2 mV·ms	61.9 ± 30.1 μV 17.4 ± 8.4 uV·ms	0.71	196 ± 54.3^8^ CoV = 27.8%
Milner-Brown et al. 1976 [36]	N = 10	MIS^1^	-	-	-	163 ± 84^10^ (61–351) CoV = 51.5%
Galea et al. 1991 [37]	N = 30 21–56 y	CIS	-	-	-	131 ± 45^8^ CoV = 34.3%
Barkhaus et al. 1990 [38]	N = 24, 51 y 22–89 y	STA	-	-	-	48 ± 9.7 (25–63), < 60 y CoV = 20.2%
Zheng et al. 2020 [26]	N = 42 50.4 y	MPS	6.2 ± 1.7mV	53.3 ± 11.8 uV	0.86	122.1 ± 48.6^9^ CoV = 39.8%
Albrecht et al. 2004 [39]	N = 10 (5♀) 18–25 y	AMPS	28 ± 7.9 mV·ms	70 ± 48.5 uV·ms (median ± SD)	-	411 ± 99^10^ (median) (232–530)
Neuwirth et al. 2011 [21]	N = 15 < 60 y	MUNIX	7.3 ± 3.9 mV	-	-	108.2 ± 51.4 (22–247) CoV = 47.5%
Zheng et al. 2020 [23]	N = 44 (23 ♀) 44 ± 10.7 y	MUNIX	6 ± 1.9 mV	MUSIX^5^ 63.5 ± 14.5 uV	1.05	97.5 ± 37.3 CoV = 38.2%
Daube 1995 [40]	N = 30	STAT	-	-	-	158 (lower limit = 58)
Aggarwal et al. 2001 [41]	N = 12 43 y	STAT	-	-	-	138 (119–169)
Murga-Oporto et al. 2003 [42]	N = 66 10–59 y	STAT	6.7 ± 1.8 mV	48.2 ± 16.5 uV	0.72	204 ± 45 (138–302) CoV = 22.0%
Slawnych et al. 1996 [43]	N = 23 (15 ♀) 33.2 ± 12.5 y 16–57 y	MUESA	-	-	-	84 ± 37 CoV = 44.8%
Henderson et al. 2007 [44]	N = 8, 52 y (36–73 y)	B-STAT^2^	-	-	-	40–58 (modal value)
Current study	N = 21 (6♀), 32.2 ± 5.9 y, 21–44 y	MScanFit	5.2 ± 1.5 mV^4^ 6.1 ± 1.6 mV	67.6 ± 19.0 uV	1.32 ± 0.31	78.5 ± 15.2 (43–103) CoV = 19.4%

Values are group means ± SD and/or ranges, unless otherwise indicated; N = number of subjects; ♀, number of females where reported; Age, in years (y). Cells with unfilled values indicate the parameter was not reported. Presented values based on stimulation of the peroneal nerve at the ankle unless otherwise indicated.

AMPS, adapted multiple point stimulation; CMAP, compound muscle action potential; CIS, computer-assisted incremental stimulation; MIS, manual incremental stimulation; SMUP, surface-recorded motor unit potential; MUESA, Motor Unit Number Estimation based on Stochastic Activation; MPS, multiple point stimulation; STA, spike-triggered averaging; STAT, statistical (Poisson); B-STAT, statistical (Bayesian); MUNIX, motor unit number index; CoV, coefficient of variation (%).

^1^Alternation-corrected MIS

^2^ Regarding the controls and patients examined in their study, the authors stated, “All the subjects had EMG evidence of denervation in muscles supplied by the nerve studied and in these subjects the nerve conduction studies were normal apart from the CMAP amplitude”

^3^ Estimated by us based on the presented means for SMUP peak-to-peak amplitude (0.03 mV or 30 μV) and mean normalized SMUP (0.55%). Thus, 0.03 mV/x = 0.0055, therefore, x = 0.03/0.0055 = 5.4 mV (peak-to peak)

^4^Stimulation at the knee (negative peak amplitude)

^5^ MUSIX (motor unit size index) = baseline-to-peak amplitude of maximal CMAP/ MUNIX

^6^ Only one study reported the actual %CMAPmax (McComas et al. 1971), whereas we calculated the values for the other studies using their presented numerator and denominator means, % CMAPmax = mean SMUP amplitude (area)/maximum CMAP amplitude (area) x 100

^7^CMAP peak-to-peak amplitude/mean SMUP peak-to-peak amplitude

^8^CMAP area/mean SMUP area (negative and positive phases)

^9^CMAP negative peak amplitude/mean SMUP negative peak amplitude

^10^CMAP area/mean SMUP area (negative phase).

Mean MUNEs across studies vary up to ~10-fold (Table 2). MUNE in the present study (78.5 motor units) is at the low end compared to the earliest methods; 200 units based on manual incremental stimulation [4, 29], 130 units based on computer-assisted incremental stimulation [37], or 122 units according to multiple point stimulation [26].

However, our MUNE is close to the mean of 84 units based on a decomposition method that purportedly better addresses the problem of alternation (i.e., activation of different axons with overlapping motor thresholds) [43]. Our mean MUNE is also similar to values derived using MUNIX (about 100 units) [21, 23]. In general, methods that are designed to account for alternation like the present study, or other statistical-based approaches, tend to produce lower MUNE compared to other methods.

Given that Table 2 data was largely recorded in healthy young adults, the smaller MUNE in the current study compared to many others may be mostly explained by the different methodologies employed. However, an apparent 3-fold range in mean MUNE across different studies does not seem to be explained by differences in CMAP maximum. Thus, mean MUNE was 78.5 (current study), 97.5 (MUNIX) [23], 122.1 (multiple point stimulation MUNE) [26], and 204 (statistical MUNE) [42], whereas the corresponding mean maximum CMAP negative peak amplitude was 5.2 mV, 6.0 mV, 6.2 mV, and 6.7 mV, respectively. This comparison suggests that study differences in mean MUNE were related mostly to differences in the derived SMUP size. Indeed, there is a strong inverse relationship between mean normalized SMUP amplitude (%CMAPmax) and mean MUNE when means from different studies are plotted together (R^2^ = 0.90, S5 Fig in S1 Appendix). This observation raises the often discussed issue of which MUNE method produces the most representative SMUP size [2, 19].

The ability of a particular approach to detect significant differences in MUNE between groups depends in part on the variability of MUNE within each group. In the current study, MUNE coefficient of variation (CoV%, MUNE SD/MUNE mean x 100) was only 19.4%, lower than 13 other studies (mean, 34.2 ± 9.7%, range 20.2–51%) presented in Table 2. Note that CoV% was not correlated with MUNE when the mean values from each of the 13 studies and the current one were plotted together (R^2^ = 0.05, N = 14). We suggest that optimizing EMG electrode position and the unique approach of MScanFit helps to lower inter-subject MUNE variability.

### 4.4 Limitations

This study has a number of limitations. Needle EMG examination of the EDB was not done. Thus, we are uncertain whether denervation (i.e., positive sharp waves and fibrillation potentials) was ongoing, nor it’s potential impact on MUNE, in our subjects [45]. Although our MUNE data may serve as reference values for healthy young adults, more persons would need to be studied (particularly persons older than 60 y) to produce normative data. Also, further experiments are necessary to compare EDB MScanFit with other MUNE methods, and to examine reproducibility and sensitivity at detecting motor unit loss, although these issues were addressed previously in different muscles [8–10, 22].

### 4.5 Conclusion

We confirmed that the EDB CMAP maximum is highly sensitive to the position of the active EMG electrode. Healthy male and female adults tolerated EDB MScan recordings when evoked by peroneal nerve stimulation at the fibular head. There was an approximate 3-fold range in MUNE and maximum CMAP amplitude across subjects, and the former was unrelated to the latter. Mean SMUP amplitude and maximal CMAP were strongly correlated, whereas there was a modest negative correlation between SMUP amplitude and MUNE. Optimized CMAP maximum together with MScanFit recordings provide a powerful combination for estimating the number and size parameters of EDB motor units.

## Supporting information

S1 Appendix EDB evoked responses and relationship between MUNE and SMUP amplitude (% CMAPmax). (PDF)
