# Supplementary material for: Estimation of the number of motor units in the human extensor digitorum brevis using MScanFit
Source: PLoS One. 2024 Apr 26;19(4):e0302214. doi: 10.1371/journal.pone.0302214 (PMC11051589; doi:10.1371/journal.pone.0302214)
Supplement: S1 Appendix — (PDF) [file pone.0302214.s001.pdf]

Supporting information for:

Estimation of the number of motor units in the human extensor digitorum brevis using MScanFit

Cliff S. Klein, Hui Liu, Yuan Xiong

This PDF file includes:

S1 Fig. Localizing the end-plate zone in the extensor digitorum brevis (EDB).

S2 Fig. Evidence indicating presence of an accessory deep peroneal nerve in 1 subject.

S3 Fig. EDB CMAP map in 1 male subject,

S4 Fig. EDB CMAP map in 1 female subject.

S5 Fig. The relationship between mean estimated number of motor units and mean normalized SMUP amplitude (% CMAPmax) in the young adult EDB, derived from reports that employed different MUNE methods.

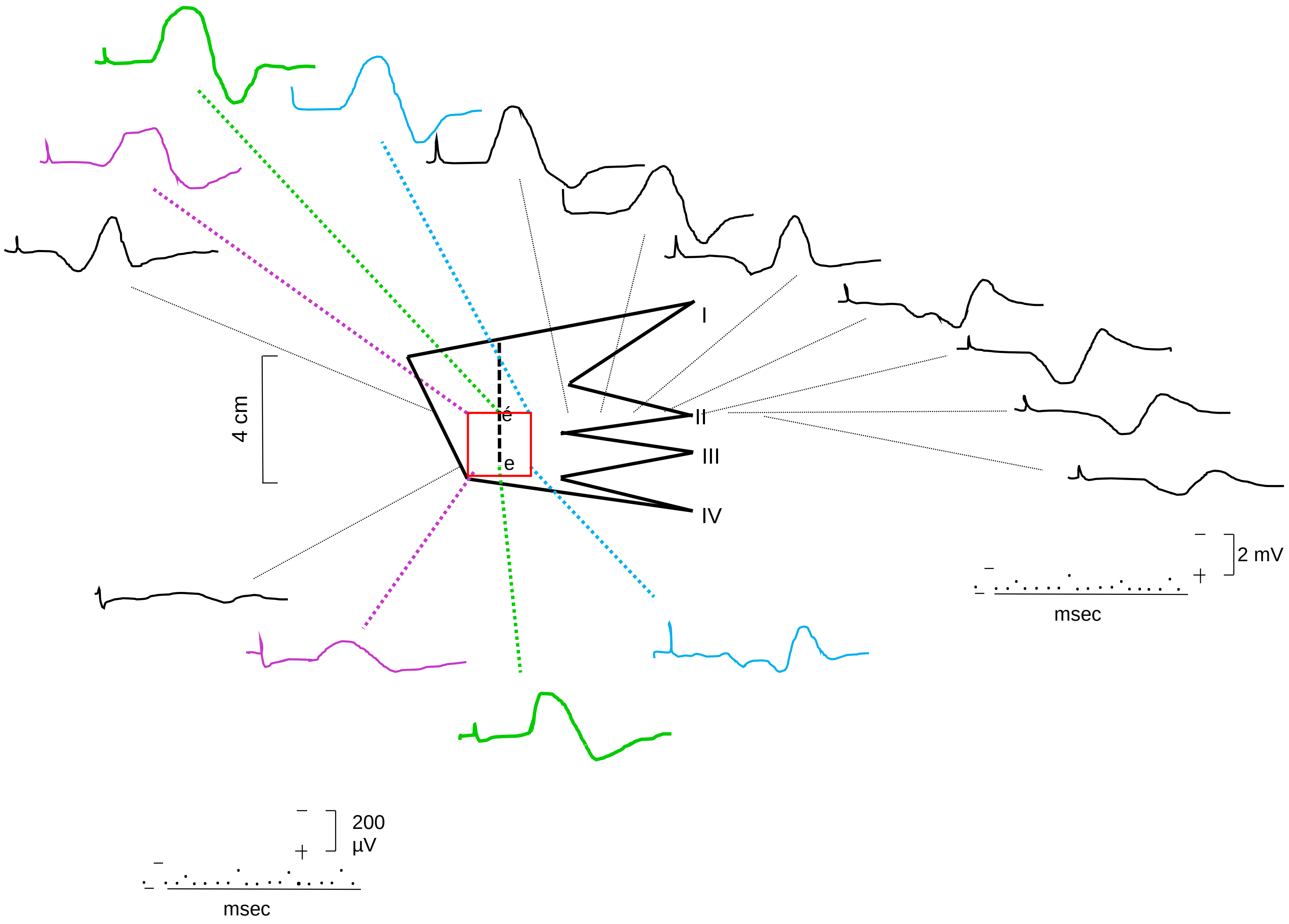

S1 Fig. Localizing the end-plate zone in the extensor digitorum brevis (EDB). This figure (including calibration scales) is a reproduction (traced) of Figure 6 from the original MUNE paper (McComas et al., 1971). However, we have reoriented it to match the alignment of the foot presented in Figure 1 of the present study and added some color. Also, we have added a 2 cm x 2 cm red square to approximate the region of the EDB that was CMAP-mapped in the current study.

Shown are potentials recorded by a silver ball electrode at different sites in the long axis of the EDB following stimulation of all motor units (maximum CMAPs- top row of waveforms) or a single motor unit (bottom row of waveforms). The largest CMAP amplitude and largest SMUP amplitude (green waveforms) were located along a zone, about 1-2 cm from the posterior muscle border (point's é and e, on the dotted line, respectively) as previously described by McComas. This end-plate zone may correspond to a zone running through the approximate mid-square of the present study. The calibration scale on the bottom refers to the SMUPs and the calibration scale on the right refers to the maximum CMAPs.

In this example, suboptimal placements of the active strip electrode by ~ 1 cm would have changed the maximum CMAP/mean SMUP peak-to-peak amplitude ratio and thereby the MUNE value. Thus, placement 1 cm distal to the end-plate zone would have increased MUNE by 1.3 times (blue waveforms), or placement 1 cm proximal to the end-plate zone would have increased MUNE by 1.17 times (purple signals). This electrode placement effect was calculated by the present authors according to the displayed calibrations, assuming the displayed SMUPs represent the average SMUP peak-to-peak amplitude at the different sites.

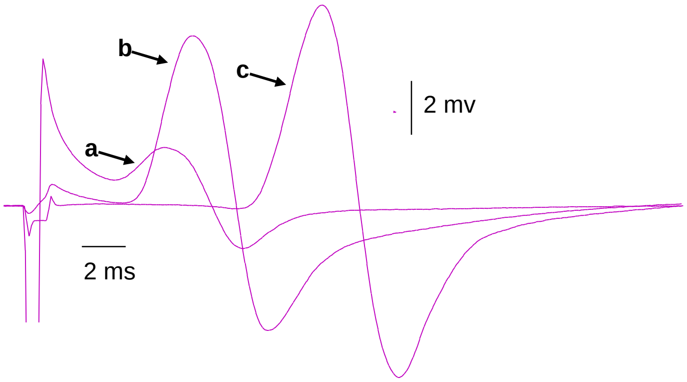

S2 Fig. Evidence indicating presence of an accessory deep peroneal nerve in 1 subject (No. 3, male, 26 y, 170 cm). The maximal EDB CMAP amplitude was smaller when evoked by nerve stimuli at the ankle (**b**, 6 mV) compared to stimuli at the fibular head (**c**, 7.3 mV), the opposite of the normal pattern. This opposite pattern suggests the presence of an accessory deep peroneal nerve. Indeed, supramaximal stimuli posterior to the lateral malleolus evoked an EDB CMAP (**a**, 1.3 mV).

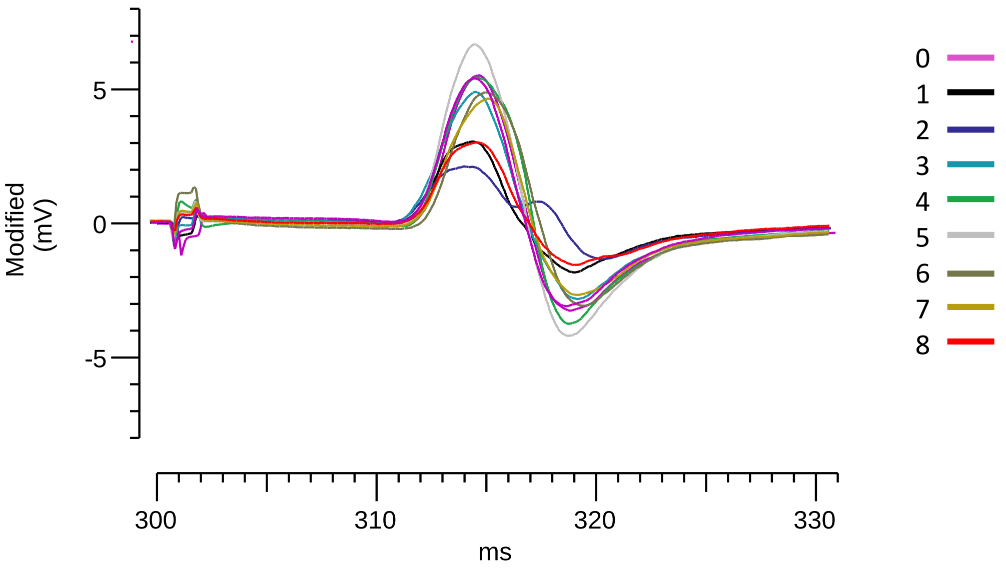

S3 Fig. EDB CMAP map in 1 male subject (no. 7, male, 33 y, 170 cm). Single maximum CMAPs from sites 0 to 8 are displayed. The largest CMAP occurred at site 5. A repeat recording was made at site 0 and is also displayed. At site 0, the CMAP amplitude of the first recording (5.6 mV) was 105% of the repeat recording (5.3 mV).

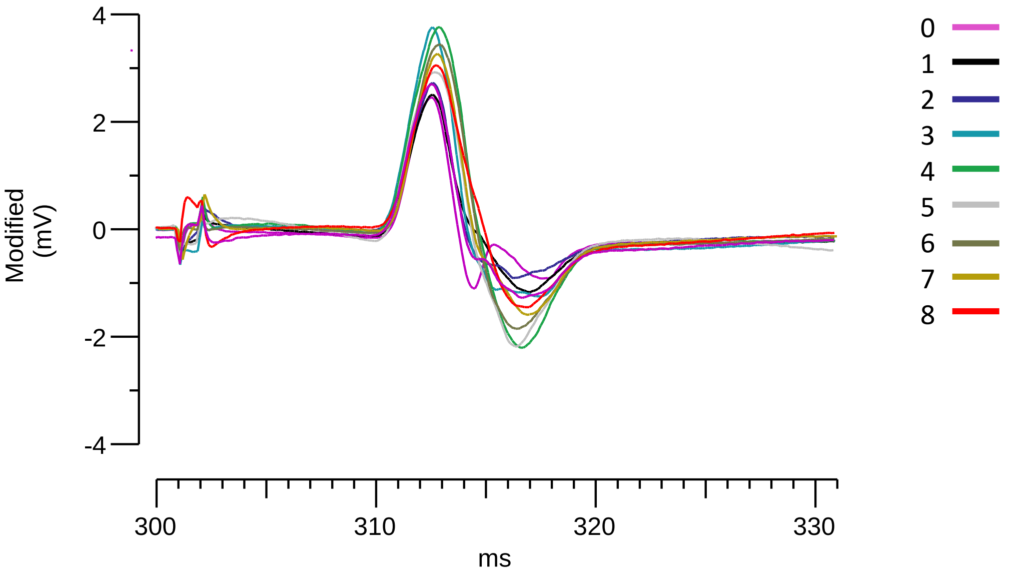

S4 Fig. EDB CMAP map in 1 female subject (no. 19, female, 37 y, 156 cm). Single maximum CMAPs from sites 0 to 8 are displayed. The largest CMAP occurred at sites 3 and 4. A repeat recording was made at site 0 and is also displayed. At site 0, the CMAP amplitude of the first recording (2.6 mV) was 93 % of the repeat recording (2.8 mV).

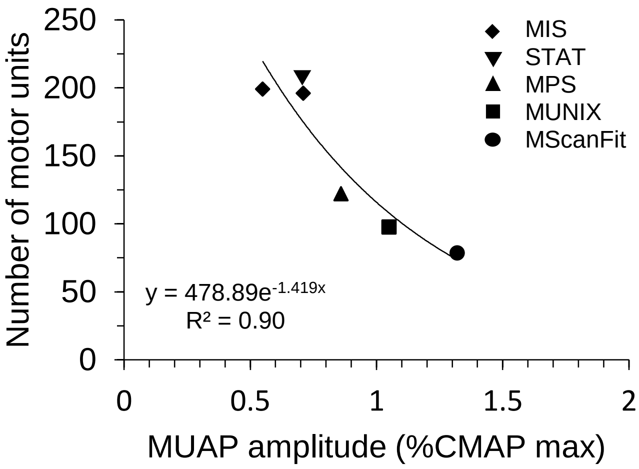

S5 Fig. The relationship between mean estimated number of motor units and mean normalized SMUP amplitude (% CMAPmax) in the young adult EDB, derived from reports that employed different MUNE methods. Data points for the 6 studies are taken from Table 3. MIS, manual incremental stimulation (McComas et al., 1971; Weir et al., 1980); STAT, statistical MUNE (Murga Oporto et al., 2003), MPS, multiple point stimulation (Zheng et al., 2020b); MUNIX, motor unit number index (Zheng et al., 2020a); MScanFit (current study, N = 21).

References

McComas AJ, Fawcett PR, Campbell MJ, Sica RE. Electrophysiological estimation of the number of motor units within a human muscle. J Neurol Neurosurg Psychiatry 1971;34:121-31.

Murga Oporto L, Menéndez-de León C, Bauzano Poley E, Núñez-Castaín MJ. [Statistical (Poisson) motor unit number estimation. Methodological aspects and normal results in the extensor digitorum brevis muscle of healthy subjects]. Rev Neurol 2003;36:601-4.

Weir AI, Hansen S, Ballantyne JP. Motor unit potential abnormalities in multiple sclerosis: further evidence for a peripheral nervous system defect. J Neurol Neurosurg Psychiatry 1980;43:999-1004.

Zheng C, Chen Z, Zhu Y, Lyu F, Ma X, Weber R, et al. Motor unit number index in quantitatively assessing motor root lesions and monitoring treatment outcomes in patients with lumbosacral radiculopathy. Muscle Nerve 2020a;61:759-66.

Zheng C, Yu Q, Shan X, Zhu Y, Lyu F, Ma X, et al. Early Surgical Decompression Ameliorates Dysfunction of Spinal Motor Neuron in Patients With Acute Traumatic Central Cord Syndrome: An Ambispective Cohort Analysis. Spine (Phila Pa 1976) 2020b;45:E829-e38.
